# Mitochondrial DNA inference between European populations of *Tanymastix stagnalis* and their glacial survival in Scandinavia

**DOI:** 10.1002/ece3.756

**Published:** 2013-09-15

**Authors:** Augustine Arukwe, Arnfinn Langeland

**Affiliations:** Department of Biology, Norwegian University of Science and Technology (NTNU)7491, Trondheim, Norway

**Keywords:** Climate change, distribution, glacial survival, immigration theories, *Tanymastix stagnalis*

## Abstract

The early observation from 1914 of *Tanymastix stagnalis* in Norway was not repeated recently, showing a rare and restricted distribution of this species. All four sampled localities were concentrated in the same area of the Trollheimen Mountains with altitudes of 900–1244 m above sea level. In March 2002, a new population of *T. stagnalis* was observed at about 50 km north of Madrid at an altitude of 1350 m. In general, all habitats with *T. stagnalis* were fishless shallow ponds and varied in size from 1 to about 300 m^2^. Natural variability of the global temperature is well accepted, but recent climate models have predicted increases in global average temperature. Based on the new biogeographical distribution, diurnal temperature variations, and biological evidence (inference with the analysis of mitochondria DNA), the immigration history of *T. stagnalis* was considered on the basis of two opposing immigration theories and in relation to the implications of global climate change. Two immigration theories, namely – the Tabula rasa and Nunatak, have prevailed in explaining the present distribution of plants and animals in Scandinavia. It was concluded that the rare occurrence of *T. stagnalis* in Norway fits into the Nunatak theory and that the species probably survived, at least, the last glaciation on Nunataks or coast refuges located in central northwestern Norway at Møre mountain and coast areas.

## Introduction

The causal mechanisms that might explain the present distribution of flora and fauna in Scandinavia have been the subject of conflicting views during the last decennium. The Nunatak hypothesis proposes that some unglaciated refuges existed in Scandinavia or elsewhere north of the southern boundary of the Pleistocene glaciers where plants and animals could survive the last or previous glacier ages (Dahl 1987). The alternative hypothesis is called the Tabula rasa hypothesis which proposes that all highly organized land plants and animals were exterminated north of the southern boundary of the Pleistocene glaciers and that the present biota all have immigrated from the south and east (Dahl 1987). Arguments for and against the controversial theories are thoroughly reviewed by several authors (Dahl 1955, 1961, 1987; Gjærevoll 1959, 1963; Brink 1966; Brochman et al. 2003). Geologists have expressed scepticism and disagreed about the extent and thickness of the ice shield during the last glacial age in Scandinavia. The possible ice-free areas have been discussed by many authors and reviewed by Sollid and Sørbel 1979; Nesje et al. 1987, 1988. Based on the distribution of block fields (a Nunatak during much of the late Weichselian glaciation probably older than 2.5 million years) and trimlines (correspond with a former glacier surface) in southern Norway and formerly existing ice sheet phases for the last glaciation in southern Norway, a new model of the Late Weichselian ice sheet was postulated (Nesje et al. 1988). This shows the possibility of an extensive distribution of unglaciated Nunataks and coastal refugia in southern Norway during the last glaciation. According to Nesje et al. (1988), the block fields were developed before the ice periods and were not covered by the inland ice sheet in Kvartær (i.e. the last 2.5 million years).

**Figure 1:**
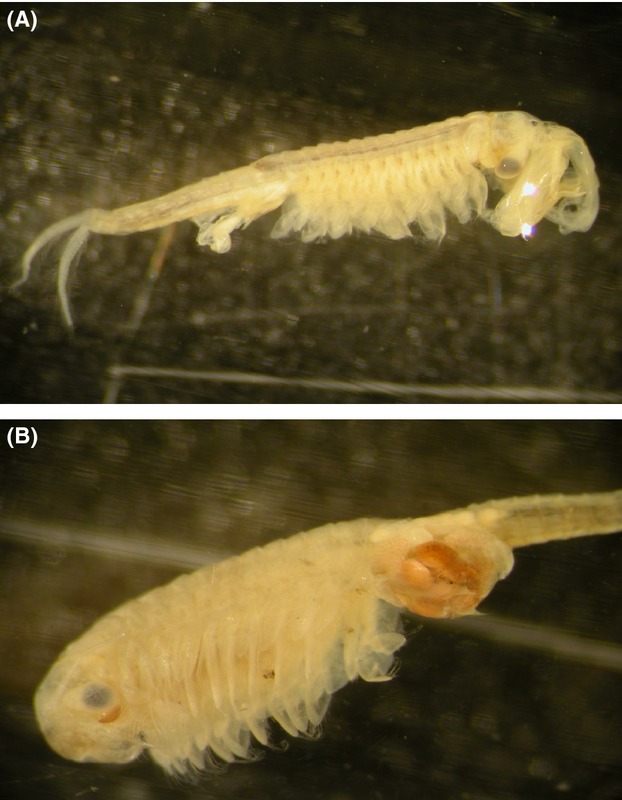
A picture of *Tanymastix stagnalis* male (A) and female (B) sampled at the Rastedam pond in 2002.

A review of the geographical distribution of European Branchiopods Anostraca (Crustacea) was given by Brtek and Thiéry (1995). Only three species of Anostraca were observed in Norway namely – the two common species *Polyartemia forcipata* (Fisher, 1851) and *Branchinecta paludosa* (Müller, 1788), and a rare species *Tanymastix stagnalis* (L., 1758; Fig. 1; Aagaard et al. 1975). *T. stagnalis* has only been observed from two localities in Norway, the Trollheimen area and North-western Norway (Gurney 1914; Engdal 1978). Two new observations of *T. stagnalis* from the same area were recently published (Langeland 2004). In contrast to *P. forcipata* and *B. paludosa* which have a northern distribution, *T. stagnalis* is distributed in southern Europe with isolated populations dispersed in Denmark, Sweden, Ireland, and Norway. In a recent study, molecular systematics and phylogeography of *T. stagnalis,* including specimens from Norway, were investigated based on mitochondrial DNA (Ketmaier et al. 2005). The aim of the present study was to investigate the phylogeographical distribution of *T. stagnalis* (Anostraca Crustacea) in Europe and to consider the causes of immigration (dispersal) and survival.

## Methods

**Figure 2:**
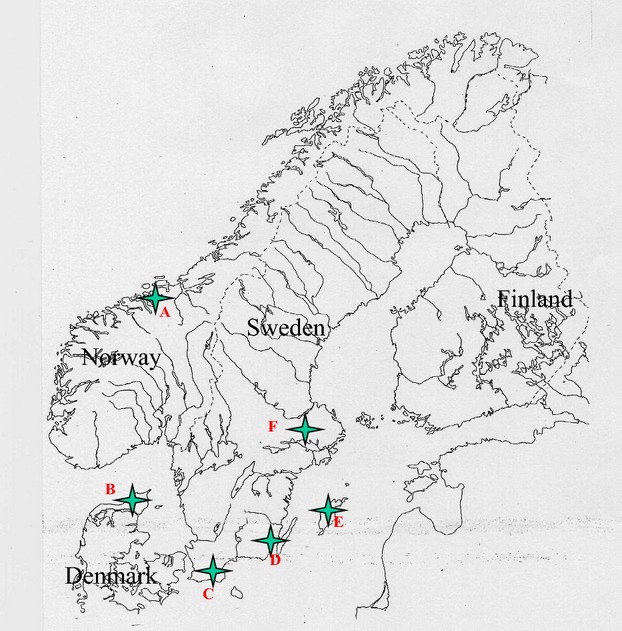
Map showing the regional (asterisk) rare distribution of *Tanymastix stagnalis* occurrence in Norway (A), Denmark (B), and Sweden (C-F). Distribution in Denmark from reference (Damgaard and Olesen 1998) and in Sweden from reference (Franzén 1996).

During July–August 1999–2010, a large number of ponds (ca. 100) were monitored to find anostracans. The ponds were located in mountains of Central Norway with emphasis on the Trollheimen area (Fig. 2). The anostracan *T. stagnalis* was recorded in four different areas in the Trollheimen Mountain area (Table 1). The localities are situated in the northwestern areas of Central Norway (Fig. 2). The ponds were studied during July and August when the animals were expected to be in post-larvae or adult stages. In an attempt to rediscover the species in the old-described location at Surnadalskluken pond (Gurney 1914), a visit to the area between July and August 2003 gave negative results. In two of the areas (Gråfjell and Rastedam), populations were recorded in three nearby ponds of different sizes. The Geithetta pond was situated at 1244 m asl (above sea level). Small amounts of brush surrounded the largest pond (i.e. Rastedam pond, Table 1). The ponds are located at the uppermost plateau of high mountains and surrounded by bare rocks more or less by shallow or deep block fields. In Spain, March 2002, *T. stagnalis* was recorded in three small pools in mountain areas of about 50 km north of Madrid City (Table 1). The localities were situated in the Guadarrama Mountains about 10 km east of Guadarrama Village, near Sta. Cruz del Valle de los Caidos and “The Franco monument and Mausoleum”. This is a new observation in Spain not previously mapped by the review (Brtek and Thiéry 1995). The Universal Transverse Mercator (UTM) – reference and altitude of the ponds are given in Table 1.

**Table 1:** *Tanymastix stagnalis* recorded in ponds in Trollheimen area Central Norway 1999–2010 and in Spain 2002.

Area	Date	Number of ponds	Altitude (m)	UTM-Reference	Conductivity (μS/cm)	Pond Size
Rastedam	May–September 1999–2003	3	904	0526436 6963767	19–24	50 × 70 m 150 cm max depth
Gråfjell	June–September 2001-2003	3	1231	0526481 6966632	12	15 × 15 m 50 cm max depth
Geithetta	August 2001–2003	1	1244	0516885 6966461	7	10 × 5 m 50 cm max depth
Levra	July 2010	1	1092	0535039 6954732	Low	20 × 10 m 40 cm max depth
Spain	10–15 March 2002	3	1350	0404360, 4500707	68	1 × 1 m 30 cm max depth

Qualitative samples of anostracans were made using a rectangular opening (20 × 30 cm) net of 0.250 mm mesh size, fastened to a rod 1.5 m long. The net was moved in a zigzag pattern along areas in the shallow pond for 10 min. All anostracans were picked out and fixed in alcohol. Visual observations were also possible on adult stages. The morphometry of the pond was described by maximum depth, length, width, and bottom substrate. The exact position and altitude of the pond were recorded using a Global Positioning System (GPS) instrument (Garmin GPS 12) making it possible to identify positions to the nearest 3–5 m. Position was recorded in UTM coordinates and plotted on topographic map. Pond temperatures were measured in surface water and in the air. Measurements of temperature every 2 h were made in two ponds in 2002 and 2003 using a temperature recorder (Hobo-H8 Onset computer corporation, Pocasset, MA). One water sample was collected and stored in cooler for later analyses of pH and conductivity using electronic pH and conductivity meters. Due to the shallow depth, all bottoms were visible over the whole pond. All ponds were photographed using a digital camera Nikon Coolpix 5000 (Tokyo, Japan). Identification of species followed the description of Fløssner (1972).

### Mitochondrial DNA (mtDNA) sequencing

Between 1999 and 2010, *T. stagnalis* collected from the different locations in Norway, Italy, Spain, Denmark, and Sweden were homogenized individually in lysis buffer (100 mM EDTA and 10 mM Tris–Hcl, pH 7.5) according to the method of Miller et al. (1988). After incubation for an hour at 55°C, 500 μL of phenol was added and centrifuged at 12,000×*g* for 10 min. Thereafter, 500 μL of chloroform was added followed by another centrifugation at 12,000×*g* for 10 min. Thereafter, genomic DNA was precipitated using ethanol, dissolved in water, and stored at −20°C until PCR analysis. Mitochondrial DNA PCR analysis for cytochrome-B was performed using primers 10612 (mtD25) and 10920 (mtD27) under the following conditions: 5 min at 95°C, 40 PCR cycles at 95°C for 30 sec, 53°C at 30 sec, and 72°C at 30 sec, followed by a final step at 72°C for min). The resulting single DNA bands were excised from a 1% low-melting agarose gel, purified, and sequenced according standard protocol.

## Results

**Figure 3:**
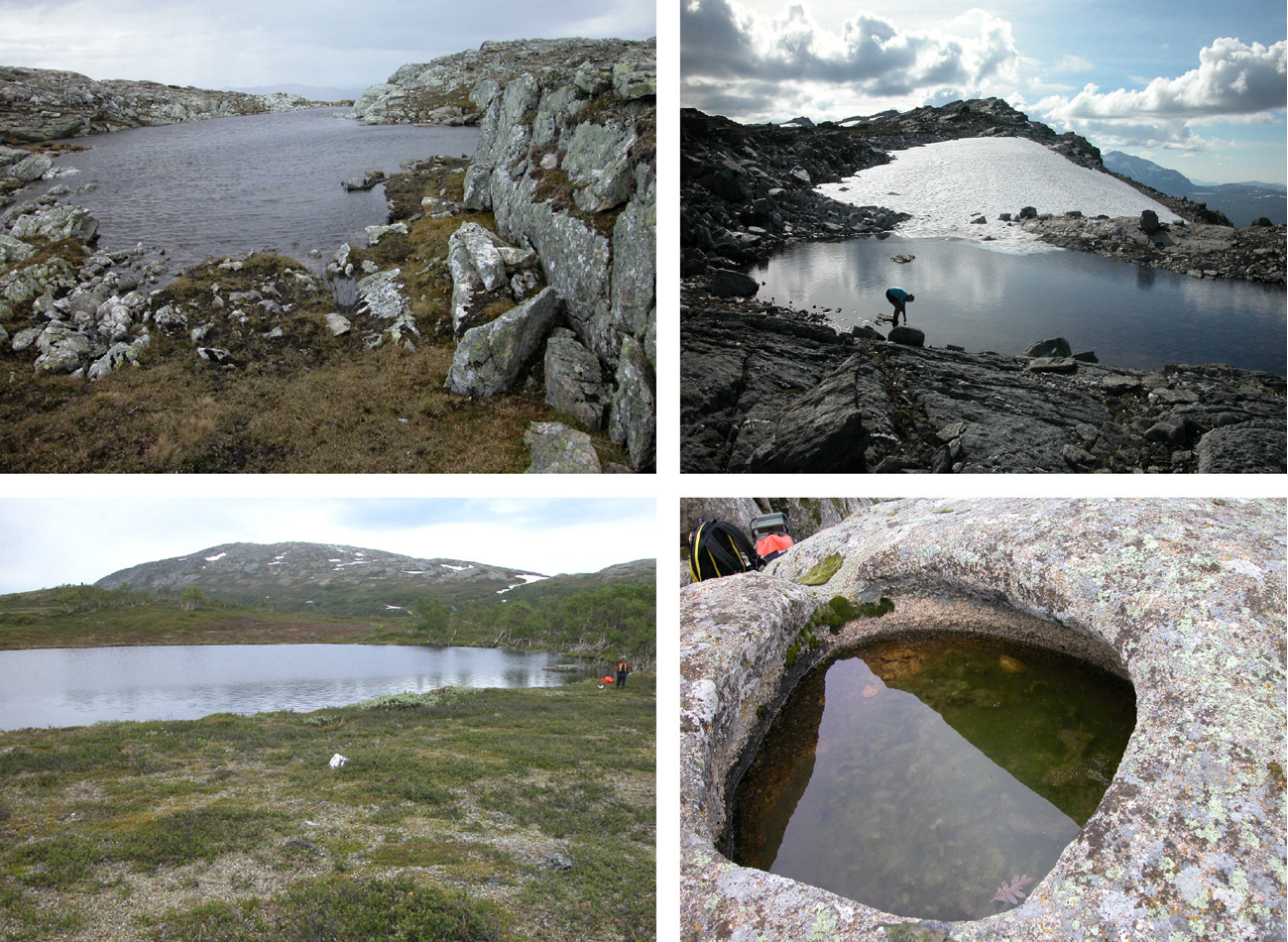
Digital photographs of *Tanymastix stagnalis* localities given in Table 1. Gråfjell pond; upper left, Geithetta pond; upper right, Rastedam pond; lower left, Spanish pool; lower right.

**Figure 4:**
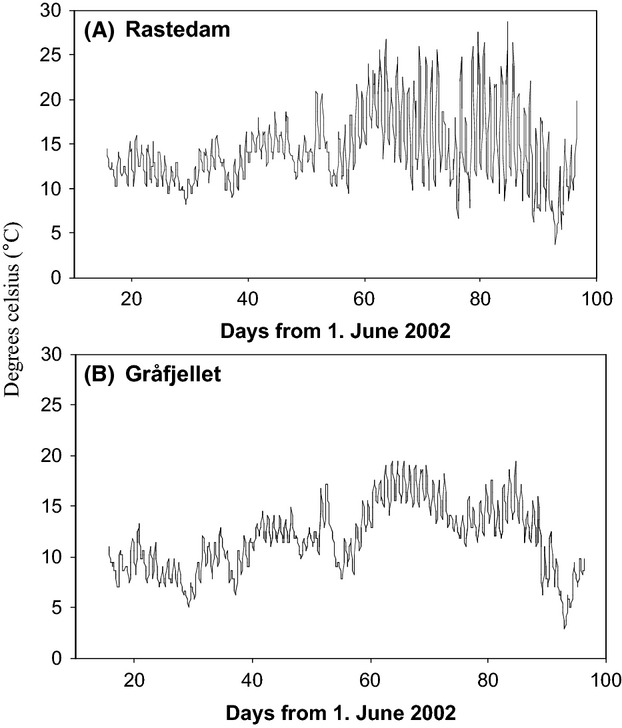
Temperature °C recordings every 2 h from June 15 to September 4, 2002 in Gråfjell and Rastedam ponds. *Tanymastix* was present during the warmest period in Rastedam and Gråfjell (i.e. present in Rastedam from May to July and in Gråfjell from June to August). None in September.

**Figure 5:**
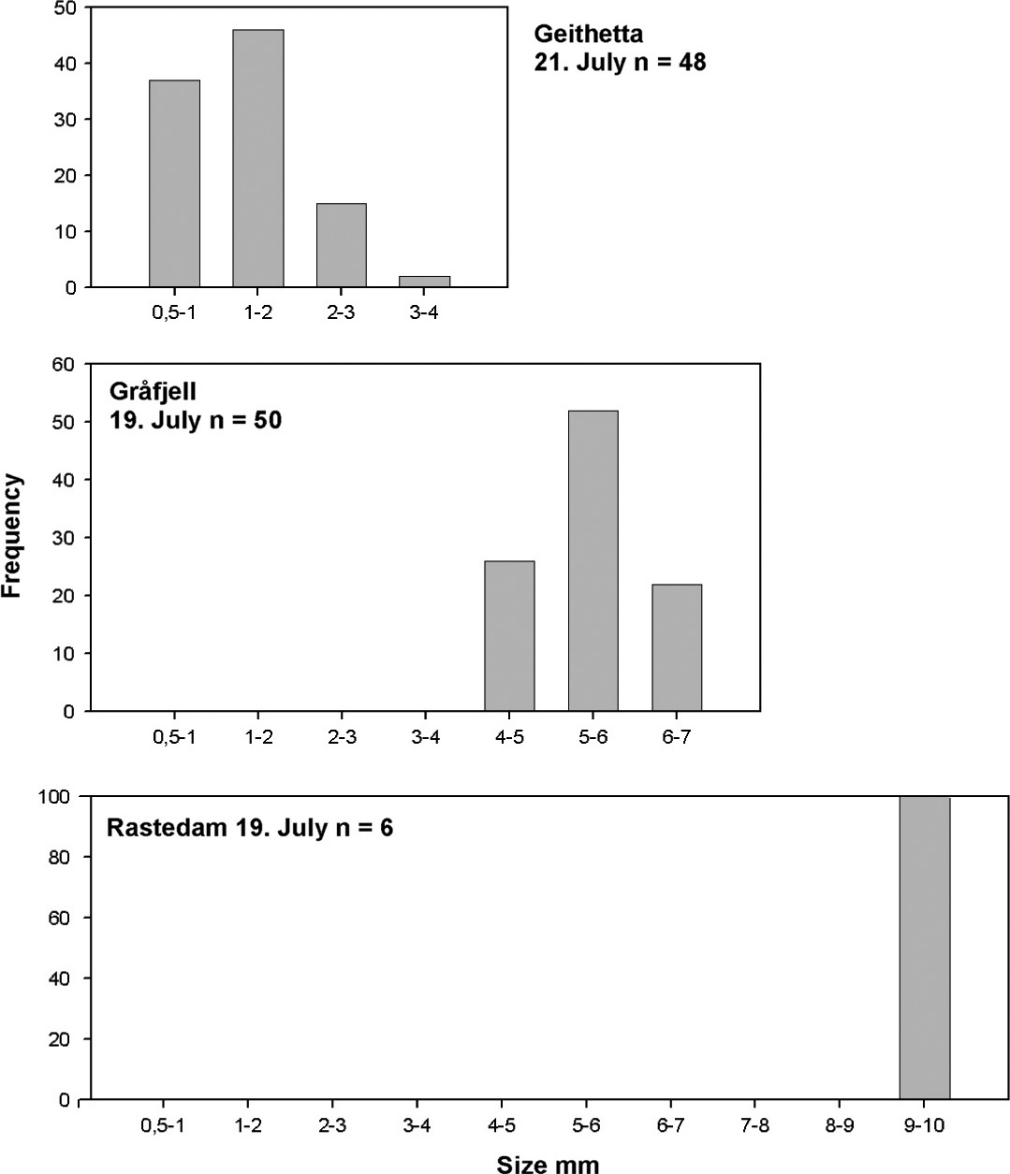
Length distribution (frequency in%) of *Tanymastix stagnalis* in the recorded ponds on July 19 and 21, 2002.

The typical habitat pattern of *T. stagnalis* is a temporary shallow pond with bare rocks at the bottom (Fig. 3) of the studied sites. Some of the largest ponds do not dry completely during autumn, and this is dependent on precipitation and temperature conditions. The ponds are all fishless and shallow. *T. stagnalis* has a wide tolerance concerning temperature as shown in Fig. 4. At daytime, the temperature may change as much as 4–5^°^C within a 24-h period. In 2002, maximum temperature of 20°C in Gråfjell pond located at 1231 m was recorded in early July. The temperature in the lower elevated pond 900 m, Rastedam pond, was on the average 5°C higher than Gråfjell. In these ponds, the substrate is covered by only small amounts of mostly inorganic fine sediments. The broken bottom substrate of rocks, most likely serves as good hiding places for the animals against predators such as birds and freshwater invertebrates. Individuals from all populations or study sites were identified as *T. stagnalis,* and visual differences were not observed The water quality is characterized as ultra-oligotrophic, with low conductivity (7–24 μS/cm) and pH around neutral (6.5–7.2). The conductivity of the Spanish pools was higher with 68 μS/cm. The time of hatching from resting eggs during spring was in concordance with the time of snow melting. At the lowest elevated pond (Rastedam), this occurred in mid-May, followed by mid-June in Gråfjell pond and mid-July at the most extreme location, Geithetta pond. In July, the size distribution was quite different in the tree ponds investigated (Fig. 5).

### Analysis of mtDNA sequence

**Figure 6:**
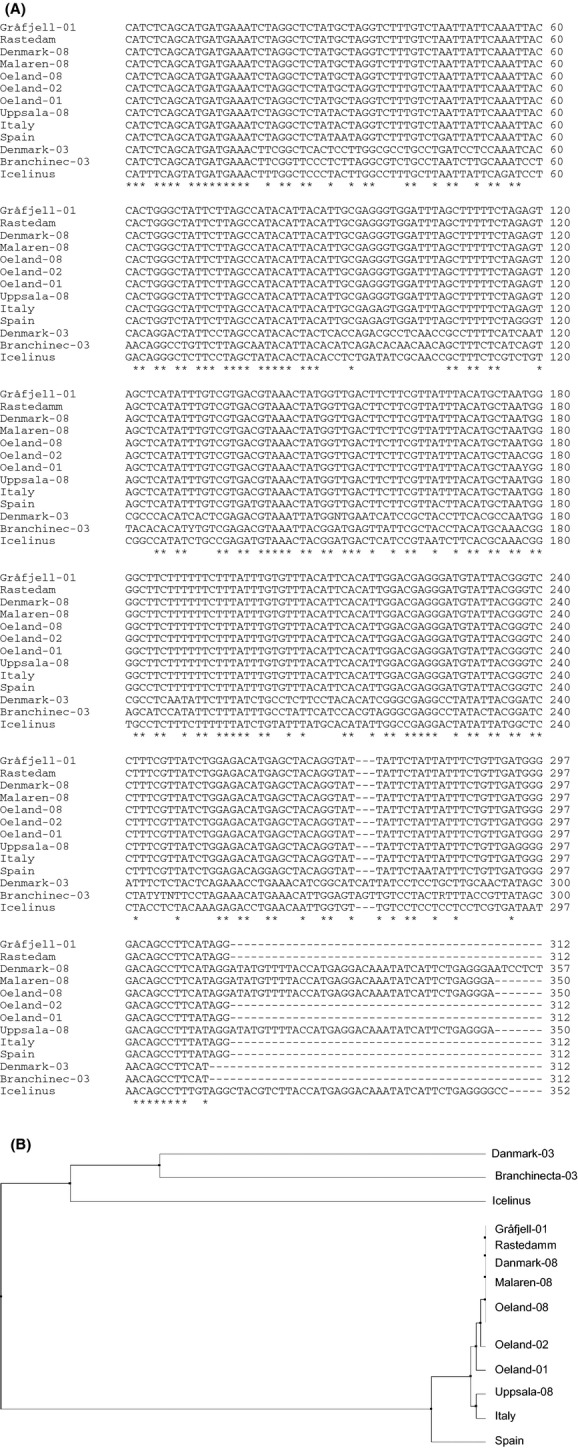
Mitochondrial DNA analyses (A) and phylogenetic analyses of the mtDNA nucleotide sequences (B) of *Tanymastix stagnalis* from Norway, Denmark, Sweden, Italy, and Spain, and *Branchinecta paludosa* from Norway.

A multiple alignment of cytochrome-b mtDNA nucleotide sequences from the studied specimens was generated using Multiple Alignment Construction & Analysis Workbench (MACAW; Fig. 6A). The cytochrome-b mtDNA sequence contained a 312-nucleotide sequence, and analysis of the nucleotide sequence showed that the Norway (Gråfjell and Rastedam) and Sweden (Uppsala and Oeland) specimens were very similar with about 6 nucleotides or 1.9% differences. Between the Italian and Norwegian specimens, there are 3 nucleotides or 1% difference, and between the Spanish and Norwegian specimens, there are 10 nucleotides or 3.2% difference. Between the Italian and Spanish specimens, there are 9 nucleotides or 2.9% difference. However, while one of the Danish specimens (Denmark 03) did not show any nucleotide similarity with the specimens from other countries, another one (Denmark 08) showed a 99% similarity with *T. stagnalis* specimens from Norway and Sweden (Fig. 6A). A phylogenetic analysis of the mtDNA nucleotide sequences of cytochrome-b from all the *T. stagnalis* specimens was performed showing that the Scandinavian specimens clustered within the same group together with the Italian specimens and a little distant from the Spanish specimens (Fig. 6B). The nucleotide sequences were aligned using ClustalW analysis and bootstrap values based on 100 samplings. This was in concordance with the results by Ketmaier et al. (2005).

## Discussion

The present observations and distribution of *T. stagnalis* indicate a restricted and rare occurrence in Norway that is far from the nearest occurrence in Denmark and Sweden (Franzén 1996; Damgaard and Olesen 1998). The first thorough sampling of freshwater crustacean in Norway was G. O. Sars pioneering work during the late 19th century (Sars 1896). He visited countless lakes and ponds all over the country and found the anostracan *Branchinecta paludosa* in few ponds around mountains of Central and North Norway, but no records of *T. stagnalis* were made during these periods. From the late 20th century, monitoring freshwater biota increased showing a total of 187 ponds where *B. paludosa* was recorded (Økland and Økland 2002). Thus, producing a bicentric distribution in Central and North Norway (Aagaard et al. 1975). Both species have similar life history and behavior, and are capable of living in fishless habitats with a wide range of physical and chemical conditions. Although large areas were not surveyed, we propose that the rare occurrence of *T. stagnalis* cannot be explained by insufficient sampling. In Norway, *T. stagnalis* has unicentric distribution similar to several plant species such as *Paper radicatum*, *Artemisia norwegica,* and *Arenaria norwegica* (Gjærevoll 1963, 1992). We did not succeed in rediscovering the population of *T. stagnalis* recorded by Gurney (1914). Eight potential ponds in the same area were visited between July and August 2003, with negative results. In one of these ponds, a large number of the Arctic Ostracod, *Eucypris glacialis*, was recorded. According to Gurney (1914), *T. stagnalis* was found together with *E. glacialis*. Thus, the occurrence of *E. glacialis* suggests that the current sampling area is the same as the pond from 1914. Two possible explanations might account for this, namely – either that *T. stagnalis* is locally extinct or that the species may have an irregular yearly occurrence as have been found for other anostracans. During a 14-year study of a temporary pond, occurrence and species composition of anostracans changed almost every year due to hatching requirements of precipitation or could be explained by a colonization-extinction hypothesis (Donald 1983). Although guided by the UTM-reference, we were not able to find the pond where *T. stagnalis* was recorded by Engdal (Engdal 1978). Habitat description of the Rastedam pond corresponds well with the same given by Engdal (1978). Personal correspondence with the author confirmed that Rastedam pond is the same as Engdal visited in 1976. The reliability of survival of freshwater animals during the glaciation is based on several preconditions – (i) the existence of refuges and (ii) special life cycle adaptations such as the ability to lay resistant eggs and hibernate in diapause stages during winter. Recent geological studies support the existence of large ice-free areas along the Norwegian coast and above the inland ice sheet as Nunataks during the last Weichselian glaciation (Nesje et al. 1987). The latter precondition is supported by our observation showing that the presence of hatching from resting eggs during spring was in concordance with the time of snow melting. At the lowest elevated pond (Rastedam), this occurred in mid-May, followed by mid-June in Gråfjell pond and mid-July at the most extreme location, Geithetta pond. Thus, the size distribution was quite different in the tree ponds investigated in the month of July (see Fig. 4).

The identification of autochthonous (rocks and sediments that have not been moved from the place where they were formed) block fields at 500 m asl on the outermost coast that rises to 1500 m asl in the interior Fjord supports the hypothesis of ice-free refuges (Sollid and Sørbel 1979). The block-field areas most probably extended above the ice sheet as Nunataks during the last maximum of the Weichselian glaciation. Thus, the block-field limit represents the upper limit of the ice sheet at that time (Sollid and Sørbel 1979). The model of the Late Weichselian ice sheet presented (Nesje et al. 1988) showed a probable existence of a large number of Nunataks in Central Norway and includes the distribution area of *T. stagnalis*. The maximum surface of the ice sheet in the Trollheimen area was 1200–1400 m above present elevation (Nesje et al. 1988). The maximum ice sheet declined westward toward the Møre coast. Studies by Grønlie (1953) indicate that the maximum elevation of the ice sheet on Trollhetta Mountain north of Geithetta in Table 1 was about 1300 m. This is about the same altitude of 1244 and 1231 m as the pond locations of *T*. *stagnalis* recorded on the neighboring mountains Geithetta and Gråfjell, respectively. The resting eggs of anostracan are dry and freeze resistant and may lie dormant for many years in temporary ponds before hatching when the ponds are filled with water the next wet season or year. Museum collection of eggs from *Lepidurus apus* was able to hatch after 28 years under dry conditions (Damgaard and Olesen 1998). The refuges could either be mountain areas rising above an inland ice, which in Inuit language is called Nunatak, or coastal refuges. When the ice melted, plants and animals immigrated into previously ice-covered land from refuges and from the unglaciated areas south and perhaps east of the inland ice. As the development time of anostracan from hatching of resting eggs to adult is very fast and takes 2–3 weeks, the ponds need to be filled with melting water only for a short period of time to maintain the lifecycle. Thus, the exceptional lifecycle adaptations of anostracan fulfill the preconditions to survive extreme conditions during cold (or warm) climate periods that probably existed in coastal or Nunatak refuges. These conditions are discussed more thoroughly below.

Recent studies indicate a continuous change of cold and warm periods during Pleistocene with a summer mean temperature near 20°C (Zagwijn 1975; Andersen and Borns 1997). After a relatively cold period during the main retreat of the North European ice sheet about 17–18,000 years ago, the late Weichselian period was very warm. The records of fossil beetles, pollen, oxygen isotopes, and the marine fauna in the North Atlantic showed that dramatic climate amelioration took place, particularly in Northwestern Europe. In Britain, the mean summer temperature increased in the order of 8–10°C. The warming period lasted from about 11–13,000 years B.P. with the last Allerød period probably as warm as the first part. Due to the warm climate, the North European ice sheet retreated rapidly at 10,000 years ago (Andersen and Borns 1997). The rapid warming of the climate probably caused difficult dispersal of the arctic flora element from the south due to high summer temperatures and strong competition from more temperate flora that immediately invaded the south Swedish areas (Dahl 1955; Gjærevoll 1959, 1992). The rate of ice retreat was up to 250 m per year north of the Middle Swedish moraines (Dahl 1955). Fossil records of tundra plants in Denmark, Sweden, and South Norway show that immigration of tundra plants seemed to have stopped far south in the mentioned areas (Gjærevoll 1992). Thus, the present distribution of mountain arctic species can hardly be explained by immigration from the south and probably survived on Nunataks or coastal refuges.

Segerstråle (1954) gave a thorough analysis of the geographical distribution and immigration history of two Gammaridae species. *Gammarus lacustris* has belonged to the fauna of Scandinavia since the last interglacial epoch. At least, it survived the last glaciation in coastal refuges of Fennoscandinavia since the warming of the ice sheet has spread from these areas far into the interior. In addition, postglacial immigration from the continent through Denmark and probably east of the Gulf of Finland has taken place. But, this stock has not yet reached the area populated from the refuges. In contrast to *G. lacustris*, *Gammarus pulex* did not survive the glacial age in Northern Europe. The species immigrated into some areas from the continent, following the retreating margin of the ice sheet, lived in the ice dammed lakes in Northern Germany and was effectively dispersed by the ice sheets and subsequent freshwater stages of the Baltic, invading many areas not reached by *G. lacustris*. Immigration into Norway was geographically possible, but probably prevented by enroute high salinity (Segerstråle 1954). Allozyme electrophoresis has confirmed the difference between populations of *G. lacustris* and an independent early colonization of the coastal region of Northwestern Norway (Vainio and Väinölä 2003). An isolated distinct stock probably survived, either along an early deglaciated coastal corridor from the southwest or directly from the ice-free continental shelf off the Norwegian coast. It is interesting to note the similarity concerning dispersal history and present distribution between the two *Gammaridae* species and *T. stagnalis*.

The occurrence of *T. stagnalis* in Denmark and South Sweden is probably a result of immigration from the south during the retreat of the ice sheet, similar to the history of *G. pulex*. The northward immigration probably stopped in southern Sweden due to rapid changes in climate and interactions with other species. In contrast, the isolated occurrence far north in Norway may have a similar history to *G. lacustris*. We therefore suggest that *T. stagnalis* possibly survived on Nunataks or coastal refuges in the Møre area in Central Norway during, at least, the last glaciation in Scandinavia. Mapping the distribution of long- and short-winged individuals of dimorphic Carabidae beetles, Lindroth (1958, 1969) concluded that the concentration of exclusively or predominantly short-winged populations in certain coastal areas of Norway could be explained only by the assumption that these were the descendants of populations that had differentiated in this direction during a period of complete isolation on glacial refugia. Of special interest is the distribution of *Bembidion aeneum* which is similar to the present distribution of *T. stagnalis* in Scandinavia. The adult active period is short in spring, and the new generation appears from the second half of July but apparently soon goes into hibernation, a prerequisite for survival on refuges during glaciation. While the short-winged morph (brachypterous flightless morph) is isolated on the central northwestern areas in Norway (similar areas as for *T. stagnalis*), both long- and short-winged morphs are found in Denmark and South Sweden along the western coast up to the southeastern parts of Norway (Lindroth 1969). Thus, the similar distribution pattern of Carabidae beetles, Gammaridae species, and *T*. *stagnalis* supports the hypothesis of survival on Nunataks or coastal refuges during at least the last glaciation in Scandinavia.

As for the early suggestions on the survival of plants on Nunataks or coastal refuges in Norway more than hundred years ago (Gjærevoll 1959, 1963; Dahl 1987), zoologists of the Nordic countries were also led to corresponding conclusions. For instance, Wahlgren (1919) proposed the existence of West-Arctic forms in the Scandinavia Lepidoptera fauna as due to survival in refuges. Lindroth (1958, 1969) postulated the same history for quite a number of Carabidae beetles. Ekman (1922) considered the Lemmings of Scandinavia as derived from such areas. This is later supported by mtDNA analysis of Norwegian and Siberian populations of lemmings (*Lemmus sibiricus)* (Fedorov and Stenseth 2001). The phylogenetic distinction and divergence estimate of 1.8% between the Norwegian and Siberian lemmings suggest that their separation pre-dated the last glaciation and implies that the Norwegian lemmings are probably relict of the Pleistocene populations from Western Europe. According to Siivonen (1982), the same reasoning could account for other mammals such as the Fennoscandian mountain Reindeer (*Rangifer tarandus tarandus*) and the eastern tundra Reindeer (*Rangifer tarandus sibiricus*) and the Norwegian shrew (*Sorex uraneus*). Analysis of genetic variation suggests that the Svalbard Reindeer must have been isolated from the Norwegian mainland Reindeer for at least 200 000 years ago (Røed 1985).

Recent studies of sediment samples from lakes at Andøya, situated on the northern coast of Norway, show that Chironomidae species, caddis flies (Insecta), and *Lepidurus arcticus* (Notostraca, Crustacea) survived on refuges during the peak of last glaciation (ca. 20,000–18,000 years. B.P.) (Solem and Alm 1994). Kverndal and Sollid (1993) stated that Nunataks plausibly existed in northern Troms, north of Andøya, during the Late Weichselian maximum. The cold-stenothermous *Apatania zonella*, (Trichoptera, Insecta), found in freshwater sediments dated back to 11–12,000 years. B.P., and other arctic/alpine freshwater invertebrates, probably colonized ice-free areas in northern Scandinavia from the east and migrated south from these ice-free areas after the last deglaciation (Solem and Alm 1994; Solem 1995). The fossil records of the circumpolar arctic species *L. arcticus* (Brtek and Thiéry 1995) showed that it could colonize Norway and Scandinavia from the north, east, and south (Solem and Alm 1994). Morphological and genetic diversity (mtDNA) analyses of *L. arcticus* by Hessen et al. (2004) revealed five distinct haplotypes with possible postglacial radiation from local refuges in North Norway, and the genetic diversity might very well be a result of past isolation events. The common anostracan in Norway, *B. paludosa,* has a circumpolar distribution with southern relicts in high mountains (Brtek and Thiéry 1995). The distribution in Norway seems to be bicentric that is similar to *L. arcticus,* with a southern occurrence in the mountains of Central Norway (Aagaard et al. 1975). During our search *for T. stagnalis*, *B. paludosa* was recorded in several localities in the same area as *T. stagnalis*. The closest locality was 3 km north of Levra location (Table 1). It is tempting to suggest that *B. paludosa*, a more commonly occurring species, survived the last glaciation in coastal refuges both in southern and northern Norway as previously proposed upon for *T. stagnalis* and other species.

A dispersal to present occurrence would be expected during retreat of the ice 10,000 years ago. The “*Tabula rasa*” is still the dominating theory in general ecological textbooks. According to Økland and Økland (1999), the geographical distribution of the inland ice sheet determines the preconditions for when and to which extent the fauna and flora could immigrate to freshwaters in Scandinavia during deglaciation. Analyses of genetic variation of 18 populations of *Saxifraga oppositifolia* indicate that the glacial survival hypothesis is superfluous to explaining the biogeographical distribution (Gabrielsen et al. 1997). The possibility still exists that *T. stagnalis* immigrated from the south during the retreat of the ice sheet and that the present distribution is a result of extinction of populations due to unfavorable environmental conditions. This seems unlikely, however, as there are large numbers of suitable ponds in the distribution gap between southern Sweden and Norway. Such areas are located along the mountain border between Norway and Sweden and in the mountain areas in Southern Norway. Most of the Scandinavian freshwater fauna, such as eastern fish and snail immigrants that are dependent on stages living in water the year round, probably migrated north and west during the deglaciation period 10,000 years B.P. As considered above, the immigration history of many animals and plants should be reconsidered. Based on new methods and investigations used in geology and biology, this general view could be considered more in accordance with new evidence. The overview presented in recent studies on past glacial history and genetic diversity of both terrestrial and freshwater species confirms the existence of refuges along the Norwegian coast and Nunataks, making it possible for many plant and animal species to survive during at least the last Scandinavian glaciation. In all groups of mammals tested, including primates, rhinoceroses, horses, rats, mice, bovids, and birds, the mean rate of sequence divergence in mtDNA is about 2% per million years (Shields and Wilson 1987; and references therein; Brown et al. 1979). Based on this divergence rate of 2% per million years (0.2 promille per 10,000 years), we did not expect any differences between the Norwegian and Swedish populations of *T. Stagnalis* for the post-glaciation period of 10,000 years after ice melting in Scandinavia (0 or <0.1 base pair in our materials). The difference between the Norwegian and Swedish populations was 1.9%; thus, a possible post-glaciation immigration hypotheses is rejected. In general, analyses of mtDNA and morphometrical data on populations of *T. stagnalis* from Norway, Spain, Italy, Denmark, and Sweden presented here provide solid support for the evolution history of the species.

The survival in high ultraoligotrophic mountain ponds demand a different adaptation in contrast to the survival in lowland productive ponds in Denmark (Damgaard and Olesen 1998), Southern Sweden, Germany, and Irish locations concerning abiotic factors as temperature and water quality. In his early fundamental work on *T. stagnalis*, Lundblad (1921) commented on the rare occurrence in the Møre area: “The Norwegische fundort beansprucht übrigens ein besonderes interesse, weil *Tanymastix* hier oberhalb der baumgrenze gefunden wurde. Die meisten übrigen fundorte liegen in der ebene”. Generally, *T. stagnalis* is considered to be a cold stenothermal species (Fløssner 1972). More recent studies, however, indicate that the species rather should be considered warm stenothermal (Mura and Zarattini 2000). Thus, the difference in adaptation between high mountain ponds in Norway and lowland productive habitats in Sweden and Denmark could be explained by difference in life history and a long time segregation between populations.

Human activities generally contribute to climate change through the emissions of carbon dioxide (CO_2_), the main gas responsible for climate change, as well as other “greenhouse” gases. Climate change is often viewed as a phenomenon that will develop in the coming century, but its effects are already being seen. The two most discussed and prominent effects of climate change are increased temperature (McNeil and Matear 2006; Achutarao et al. 2007; Hoegh-Guldberg et al. 2007) and carbon dioxide (CO_2_). Herein, our study ponds are all fishless and shallow, and *T. stagnalis* was shown to have a wide temperature tolerance. For example, daytime temperature may change as much as 4–5^°^C within a 24-h period in our study ponds. In 2002, maximum early July temperature of 20°C was recorded in the Gråfjell pond which is located at 1231 m. The temperature in the lower elevated pond of 900 m – Rastedam pond was on the average 5°C higher than Gråfjell. Unfortunately, until now, very few studies have investigated these effects of climate change or provide ecological, biogeographical variables of these changes on coastal marine and high mountain ecosystems, and the data that do exist suggest that delicate early life-history stages may be particularly susceptible. However, there are opinions that these stages might have an inherent plasticity that allows them to deal with certain levels of environmental stress. Therefore, the restricted biographical distribution of *T. stagnalis* may provide valuable evidence on how global climate change may affect species diversity and adaptability.
